# The Book World of Medicine and Science

**Published:** 1899-07-15

**Authors:** 


### The Book World of Medicine and Science.

Pritchard's London and Londoners, 1899: What to See, What to Know, What to Do, Where to Shop, and Practical Hints. Edited by Rosalind Pritchard. (London: The Scientific Press. 1899. Price 2s. net.)

We noticed this little handbook to London and all its ways in favourable terms last year, and we have nothing very different to say about the new edition which has now come out. It tells all that a certain class of people want to know, and no doubt it also omits a good deal—of what they do not want to know. Hence perhaps one of its chief claims to the attention of comfortable travellers, who like even a guide-book to be well bound in handsome blue leather, who want to know what sort of shops to go to, where to dine, how to dress, how to word their invitations, where to spend their mornings, their afternoons, and their evenings, and even where to go at the week-end, and who, above all things, wish to do while in London as Londoners do. In fact, it is a well-to-do guide to well-to-do London, for the use of well-to-do people. The book is nicely printed, its pages are so arranged as to show up very clearly the various topics dealt with, and it will prove, we should think, a very useful guide to many visitors. Perhaps the weakest point about it is the lists of shops, some of which, for an enormous place like London, are obviously somewhat short. It is amusing to find the name of only one chemist given, while those of the doctors cover many pages. Talking of doctors, we confess we do not quite like the long string of names of medical men, with their addresses, all sorted out according to their assumed lines of practice. We see some names whose owners we feel quite sure cannot have assented to their being used in such a manner. Still, we have no doubt that this is just the sort of information that the readers of a guide-book like to have.

Medical Gymnastics. By Anders Wide, M.D. Pp. 372. Ninety-two illustrations. (London: Sampson Low, Marston, and Co. 1899.)

Although medical gymnastics were known long ago, we owe their modern introduction almost entirely to Peter Henry Ling, a Swede; and as early as 1813 and 1837 Stockholm could boast of two medical institutions for their practice and their study. Certainly it is not a very pleasant reflection that, while some of the medical sciences have been elaborated at our schools to an extent wholly unnecessary for the medical practitioner, balneology, massage, and medical gymnastics are practically ignored, and the student or young practitioner is left to pick up a knowledge of these important adjuncts to medical treatment as best he can, or, perhaps, never to pick it up at all. It is equally humiliating to think that the man who wishes to understand these subjects has to read more foreign and American works than English ones. It is a somewhat common notion that expensive appliances have to be provided for these medical gymnastics, whereas none are absolutely essential. In fact, the treatment has often to be carried out at the patient's home where apparatus cannot be obtained at all. But if appliances be unnecessary, much practice is needed to become a skilled operator. Before being examined the Swedish expert has to go through a three years' course of training, and for medical students the course is of one year's duration. The term "Swedish gymnastics" includes a considerable proportion of "massage." About 140 pages of the volume before us are taken up by descriptions of the various "movements," and these are not only well given but clearly illustrated. When we read the chapter dealing with the practical application of gymnastics we are at once struck with the care exercised by the Swedish physicians in prescribing these "movements"; how every condition of the patient is considered, and how well is pointed out the absurdity of supposing that in medical gymnastics we have the universal remedy. Evidently Dr. Wide has no sympathy with those optimists who think, or profess to think so. Diseases of the circulatory system have 50 pages allotted to them, and assuredly it is to this section that most medical men will first turn, because, in England at least, the treatment is comparatively new for these diseases, and it upsets the theories instilled into us in the days of our studenthood. Early in this chapter we are warned that the treatment must continue "a long time and be frequently repeated; but this will be the case, too, with all other remedies for heart diseases. Gymnastic treatment has, however, the advantage that its effect will be more lasting than that of any other." Of course, the principle of the treatment is that the work of the heart is facilitated by improving the weakened circulation, and as the "passive movements" of "kneadings," "rollings," and "respiratory movements" lessen the venous congestion, we have at once an explanation of the benefit derived by heart cases. The clinical observations of Astley Levin on 6,000 pulses are quoted, and the table given shows us that the Swedish movements mentioned above reduce the frequency of the pulse by from eight to ten beats a minute. Dr. Wide thinks that only "passive mevements" should be used at first in heart disease, and that "active movements" should follow later. Oertel's terrain-kur is referred to, and believed in, especially in fatty infiltration; but it is strange that no mention is made of the Schott treatment. The book seems to have been printed in Stockholm, but it is singularly free from typographical errors. In fact, we noticed only one—"benefitted" being spelled with two "t's." The work has gone through two editions in Germany, and there is one French.

The Pocket Pharmacopœia. By Frederick Hudson Cox, F.C.S., and J. Stokes, M.D. (London: Baillière, Tindall, and Cox. 1899. Pp. 200. Price 3s. 6d.)

This little volume is the second edition of a work written by the late Dr. Armand Temple. It consists essentially of an epitome of the British Pharmacopœia (1899), to which is added some account of therapeutical actions and of the natural orders and active principles of the vegetable substances therein contained. A rather formidable list of errata is printed on an early page, and we have a shrewd suspicion that others are to be found. The information given respecting "therapeutical action" is often trite and sometimes ambiguous. Aconite root is, we are told, "used in febrile states and in dropsy and neuralgia." The book may prove useful to students preparing for certain examinations, but will not, we imagine, supplant any of the recognised textbooks.

